# Enhanced Production of Tetramethylpyrazine in *Bacillus licheniformis* BL1 through aldC Over-expression and acetaldehyde Supplementation

**DOI:** 10.1038/s41598-020-60345-3

**Published:** 2020-02-26

**Authors:** Wu Meng, Feng Ding, Rui-Ming Wang, Teng-Fei Wang

**Affiliations:** 1 State Key Laboratory of Biobased Material and Green Papermaking (LBMP), Qilu University of Technology (Shandong Academy of Sciences), Jinan, Shandong 250353 People’s Republic of China; 2 Key Laboratory of Shandong Microbial Engineering, Qilu University of Technology (Shandong Academy of Sciences), Jinan, Shandong 250353 People’s Republic of China; 3 Key Laboratory of Pulp and Paper Science & Technology of Ministry of Education, Shandong Province, Qilu University of Technology, Jinan, Shandong 250353 People’s Republic of China

**Keywords:** Applied microbiology, Metabolic engineering

## Abstract

*Bacillus licheniformis* BL1 was used as a starting strain to construct the recombinant tetramethylpyrazine (TMP)-producing strains by over-expression of the α-acetolactate decarboxylase gene (*aldC*) and α-acetolactate synthase gene (*alsS*), named BLC, BLS and BLCS, respectively. Then the addition of acetaldehyde was use to enhance the TMP yield in the fermentation process. During microaerobic fermentation, the *aldC*-overexpressed BLC strain produced 43.75 g TMP/L which was 15.47% higher than the TMP in culture yielded using the initial BL1 strain. Furthermore, the acetoin yield as TMP precursor similarly rose by 23.06% in BLC recombinant strain. In contrast, the 2,3-BD increased by 23.2% in the recombinant BLCS. TMP produced by BL1 could be bolstered via the supplementation of the acetaldehyde in fermentation medium. This method also has the same effect on the BLC strain.

## Introduction

Tetramethylpyrazine (TMP), is a heterocyclic compound that contains nitrogen and has a taste similar to roasted nuts^1,2^, leading to its common use for flavor in a lot of Chinese white liquors^3^. It is also a central part of Ligusticum chuanxiong Hort, and it is always employed as a means of treating some diseases, such as cardiovascular and cerebrovascular^4^. Pharmacological studies have demonstrated the ability of TMP to inhibit platelet aggregation, mediate vasodilation, and enhance coronary blood flow. Besides, this compound is widely employed as a flavor additive in the culinary industry^5,6^, and also used as a standard compound in many other industries^7^.

TMP can be generated via chemical or biological synthesis^8,9^. Two major substrates forming pyrazines in the Maillard Model Systems are alanine and glycine. N-terminal amino acids represent both a source of nitrogen and can male up alkyl side chains in certain alkylpyrazines^8,10^^.^*Kosuge et al*.^11^ found that microbes were capable of producing pyrazine in their study of *Bacillus subtilis*. The TMP that can be detected within Chinese white liquor has similarly been shown to be of primarily microbial origin rather than from the Maillard reaction^12^. TMP is now hypothesized to be produced via a mechanism dependent upon the dynamic coupling of enzyme/thermal catalysis during solid-stage fermentation in Chinese liquor^13^. *Bacillus sp.* are capable of yielding high quantities of TMP when using acetoin as a precursor in an endogenous precursor screening strategy^9^. Such microbial fermentation can produce TMP in a more cost-effective and environmentally friendly manner than more traditional enzymatic production strategies^14^.

To our knowledge, the precursor of diacetyl is α-acetolactate. The α-acetolactate decarboxylase gene (*aldC*) can convert α-acetolactate to acetoin. Acetoin is the precursor of TMP, and acetoin can be converted with 2,3-butanediol (2,3-BD) each other. *B. lincheniformis* can commendably produce the TMP after gene modification. Through strengthening the degradation process and blocking the competing pathways, the carbon flux can flow to acetoin biosynthesis pathway. The acetoin then was accumulated to bolster TMP production. Glucose-derived TMP yields were higher for engineered strains relative to the initial controls. We additionally explored a novel means of adding supplemental acetaldehyde during fermentation, thereby enhancing TMP yields while keeping the overall cost low.

## Materials and Methods

### Cells and reagents

The strains, plasmids and their relevant genotypes for this study are compiled in Table 1. Table 2 lists all the PCR primers in this study. All DNA manipulation was conducted via standard approaches. *Escherichia coli* was cultured at 37 °C with LB medium (10 g/L NaCl, 10 g/L tryptone, 5 g/L yeast extract) plus ampicillin (100 μg/mL) for transformant selection. During acetoin and TMP fermentation, 70 g/L glucose was supplemented into the LB broth, and every 12 hours we added 5 mL of the supplement (1 mg/mL glucose). In addition, LB plates containing 100 μg/mL filter-sterilized Ampicillin antibiotic were employed for *B. lincheniformis* transformant selection of Amp resistant strains. Solid medium was prepared via the addition of 20 g/L agar.

**Table 1:** Bacterial strains and plasmids used in this study.

Strains/plasmids	Relevant characteristics	Source or reference
**Strains**		
*E. coli* DH5α	Host strain in gene cloning/General cloning and storage of plasmids	Laboratory stock
*B. licheniformis* BL1	For production of TMP	Laboratory stock
*B. licheniformis* BLS	The mutant derived from *B. licheniformis* BL1	Laboratory stock
*B. licheniformis* BLC	The mutant derived from *B. licheniformis* BL1	Laboratory stock
*B. licheniformis* BLCS	The mutant derived from *B. licheniformis* BLC	Laboratory stock
**Plasmids**		**This study**
PMA5	For construction of the gene-over expressed vector	Laboratory stock
PMA5-*alsS*	For *alsS*-over expressed	This study
PMA5-al*dC*	For *aldC*-over expressed	Laboratory stock
PMA5-al*dC*-*alsS*	For *aldC*-*alsS*-over expressed	Laboratory stock

**Table 2:** Primers used in this study.

Primer	Sequence (5′→3′)	Digestion site
*alsS*-F1	cctaaaaaggagcgatttacatatgATGAATAATGTAGCCGTA	*Nde*I
*alsS*-R2	gctagcttgagctcgactctagaggatccAGTTCTAACGAATCTCCG	*Bam*HI
*aldC*-F1	ggatcctctagagtcgagctcaagctagcATGAAAAGTGCAAGCAA	*Bmt*I
*aldC*-R2	gaatttcgacctctagaacgcgtAATGAGCCCTAACGGAA	*Mlu*I

### Recombinant strain production

**Figure 1:**
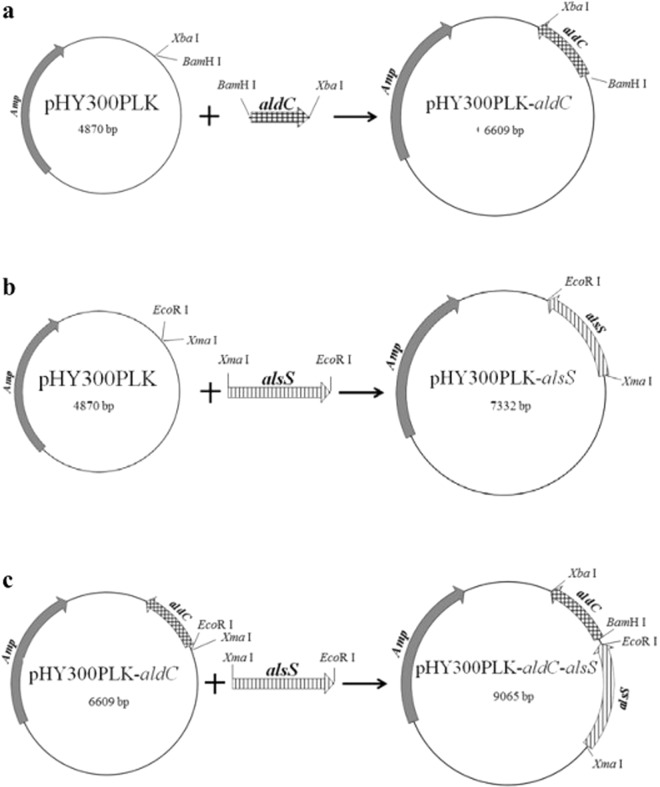
Construction of plasmid PMA5-aldC, PMA5-alsS, and PMA5-aldC-alsS. The different plasmids are depicted as (**a–c**).

The oligonucleotides listed in Table 2 were used to construct plasmids (Fig. 1) as a means of preparing a genome integration cassette. First, *B. lincheniformis* BL1 gDNA was used for the amplification of a 1739 bp CDS region of aldC and a 2462 bp CDS region of alsS using the aldC-F1/aldC-R2 and alsS-F1/alsS-R2 primer pairs, respectively. The PCR products were digested with *Eco*RV/ *Eco*RI and *Bsr*GI/ *Nco*I, respectively, and then inserted into PMA5.1 to construct the PMA5.1-aldC plasmid (Fig. 1a) and the PMA5.1-alsS plasmid (Fig. 1b), respectively. Finally, the CDS region of aldC and the CDS region of alsS were cloned into the plasmid PMA5.1 to construct the PMA5.1-aldC-alsS plasmid. The restriction sites were *Eco*RV/ *Eco*RI and *Bsr*GI/ *Nco*I (Fig. 1c).

### Production comparation of TMP production by different bacteria strains

We selected an individual *B. lincheniformis* BL1 colony that was then added to 5 mL LB with the corresponding antibiotic used for selection. Bacteria were cultured for 12 h at 37 °C and 200 rpm, then 2% (v/v) of this mixture was collected and combined for 12 h in a 250 mL flask with 50 mL LB containing 10 g/L glucose. For fermentation, a 4% (v/v) inoculum was incubated with this culture at a starting optical density (OD_600_) of 0.05 with the following parameters: a 500 mL flask containing 200 mL of LB and 70 g/L glucose was steadily mixed over 8 days at 200 rpm, and every 12 h a 5 mL volume of the glucose supplement (1 mg/mL) was added. The pH was maintained at 7.5 using 10 M NaOH solution.

### Analytical methods

Fermentation broth biomass and OD_600_ were measured via spectrophotometer (UV-722, Shanghai Xinmao Instrument Company Limited, China) at appropriate time points. An enzymatic membrane biosensor (SBA-40C, Institute of Biology, Shandong Academy of Sciences, China) with a glucose oxidase-immobilized membrane was utilized for the measurement of glucose levels in the fermentation broth. TMP levels were established via the headspace solid-phase microextraction and gas chromatography-nitrogen, as in past studies^5,15^. Acetoin and 2,3-butanediol (2,3-BD) levels were measured by gas chromatography^16,17^.

### Statistical analysis

Experiment datas were accompanied by the number of experiments independently performed and expressed as mean ± SD. The differences of the acetaldehyde supplementation and the transformants, were confirmed by the Student’s *t* test when compared with the parental strain. Differences at *P* < 0.05 were considered to be significant differences in statistics.

## Results

### Characterization of the *aldC*/*alsS* over-expressed transformants

**Figure 2:**
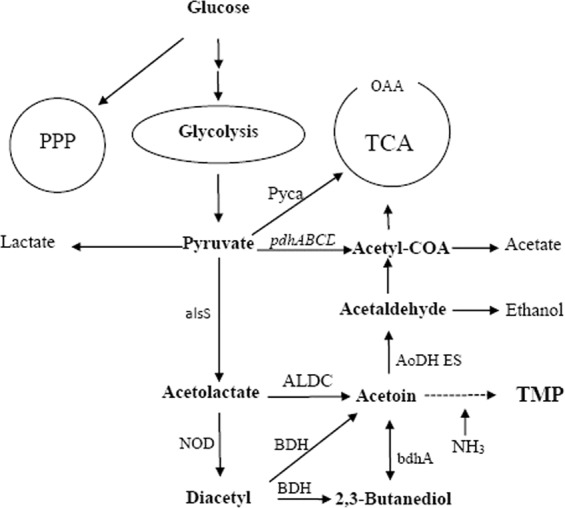
Tetramethylpyrazine (TMP) biosynthetic pathway and other overflow metabolism pathways in *B. lincheniformis* BL1. *AlsS*, *ALDC*, and *bdhA* encode acetolactate synthase, acetolactate decarboxylase, and 2,3-butanediol (2,3-BD) dehydrogenase, respectively. PPP, pentose phosphate pathway; TCA, tricarboxylic acid cycle; NOD, Oxidative decarboxylation of non-enzymatic catalysis.

**Figure 3:**
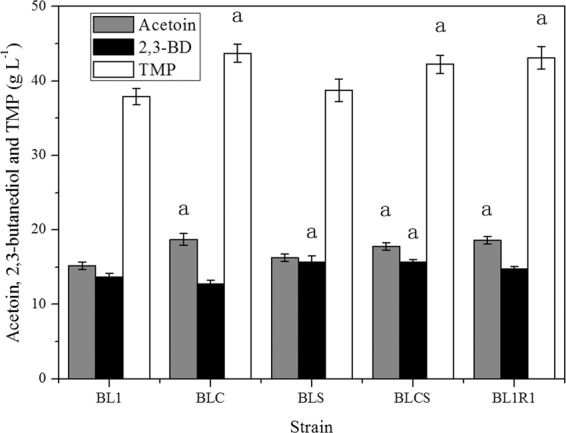
Production levels in wild-type and mutant strains. Data are average of three independent experiments and error bars represent ± SD. The maximum acetoin (gray bar), 2,3-BD (black bar), and TMP (blank bar) concentrations of BL1, BLC, BLS, BLCS, and acetaldehyde-regulated BL1 (BL1R1) cultivated with 200 ml LB in 500 ml flask shaken at 200 rpm and 37 °C for 144 h, 72(96) h, and 168 h. Data were confirmed by Student’s *t* test (^a^*P* < 0.05, n = 3).

In this study, the acetoin and acetolactate synthesis pathway was strengthened by over-expression of α-acetolactate decarboxylase gene (aldC) and α-acetolactate synthase gene (alsS), respectively. Acetoin, a tetramethylpyrazine (TMP) precursor (Fig. 2), accumulates and bolsters TMP yields. Relative to the acetoin yield of the initial strain (*Bacillus lincheniformis* BL1), that of the *aldC* and *alsS* overexpressed mutant strains (*B. lincheniformis* BLC and *B. lincheniformis* BLS) increased by 23.06% (w/w) from 15.22 g/L to 18.73 g/L and 6.77% (w/w) from 15.22 g/L to 16.25 g/L, respectively. Maximal TMP yield also rose by 15.47% (w/w) from 37.89 g/L to 43.75 g/L and 2.27% (w/w) from 37.89 g/L to 38.75 g/L in the *B. lincheniformis* BLC and *B. lincheniformis* BLS, respectively. However, 2,3-BD production fell by 6.73% (w/w) in the *aldC* overexpressed mutant (*B. lincheniformis* BLC) and increased by 14.62% (w/w) in the *alsS* overexpressed mutant (*B. lincheniformis* BLS) (Fig. 3).

### Characterization of the *aldC-alsS* overexpressed recombinant strain

Compared with the acetoin and TMP yields of the *aldC* overexpressed mutant strain (*B. lincheniformis* BLC), the acetoin and TMP yields of the *aldC-alsS* overexpressed mutant strain (*B. lincheniformis* BLCS) decreased by 4.9% (w/w) from 18.73 g/L to 17.81 g/L and 3.43% (w/w) from 43.75 g/L to 42.25 g/L, respectively. In contrast, the yield of 2,3-BD rose by 23.2% (w/w) in the *aldC-alsS* overexpressed mutant strain (*B. lincheniformis* BLCS) (Fig. 3).

### Effect of acetaldehyde supplementation in the fermentation process

**Figure 4:**
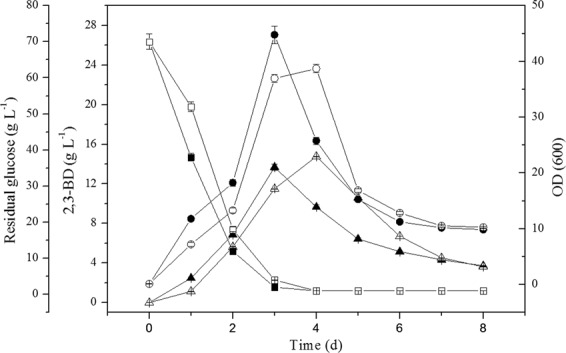
Effect of acetaldehyde addition on cell growth (●), 2,3-BD production (▲), and residual glucose concentration (■) by *B. lincheniformis* BL1. Data are average of three independent experiments and error bars represent ± SD. Product profiles of BL1 (open) and BL1R1 (filled symbols) are shown.

The effect of supplemented acetaldehyde on TMP and acetoin production in BL1 fermentation process was explored via the addition of 1, 2, 4, and 8 g/L acetaldehyde in the medium. Acetaldehyde supplementation of BL1 media improved TMP yields, with the addition of 1 g/L to 2 g/L of supplemental acetaldehyde impacting cell growth (Table 3), TMP and acetoin, the concentration of residual glucose, and the yield of 2,3-BD (Fig. 4). The yield of TMP will increase with the increase of the dosage of acetaldehyde up to a dose of 2.0 g/L acetaldehyde, after which TMP levels do not rise further (Table 3). Relative to unsupplemented BL1 (no dosage), the maximal TMP and acetoin yield rose by 13.83% (w/w) and 22.27% (w/w), respectively, in BL1R1 (BL1 with 1 g/L acetaldehyde). These increases were detected following 144 h and 168 h of culture, respectively (Fig. 3). These results suggest that a 1 g/L initial acetaldehyde concentration is ideal for maximizing the yield of TMP and acetoin in BL1.

**Table 3:** Effect of acetaldehyde addition on TMP production by *B. lincheniformis* BL1 (n = 3).

Cultivation conditions				Time^a^ (h)	Yield of Acetoin (g·L^−1^)	Time^b^ (h)	Yield of TMP (g·L^−1^)	OD_600_^max^
Initial glucose concentration (g·L^−1^)	pH	T^c^ (°C)	acetaldehyde addition (g·L^−1^)					
70.0	7.5	37	0	120.1	15.22 ± 0.24	168.5	37.89 ± 0.40	44.78 ± 0.87
			**1.0**	**144.1**	**18.61** ± **0.75**^**d**^	**168.8**	**43.13** ± **0.58**^**d**^	**38.72** ± **0.92**^**d**^
			2.0	144.8	15.25 ± 0.63	168.7	38.05 ± 0.66	30.62 ± 0.45^d^
			4.0	168.6	9.36 ± 0.41^d^	192.6	27.69 ± 0.34^d^	20.36 ± 0.33^d^
			8.0	192.8	2.41 ± 0.07^d^	192.8	3.87 ± 0.12^d^	0.85 ± 0.02^d^

^a^Time in hours from inoculation to the maximal Acetoin concentration arrived.

^b^Time in hours from inoculation to the maximal TMP concentration arrived.

^c^T, temperature.

^d^Values of the effect of acetaldehyde addition on production are significantly (Student’s t test, *P* < 0.05, n = 3) different from the parental strain BL1.

Data are average values and standard deviations of triplicate experiments.

### Effect of the acetaldehyde supplemention on the fermentation process

**Figure 5:**
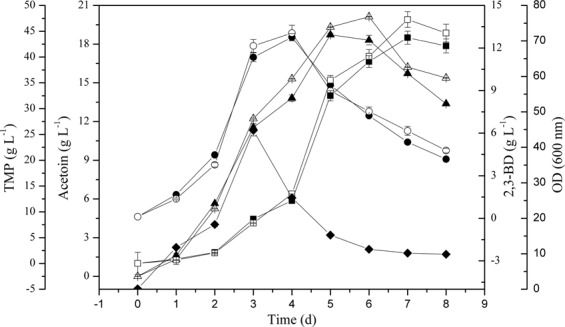
Effect of acetaldehyde addition on TMP production (□), Acetoin production (Δ) and 2,3-BD production (○) by B. lincheniformis BLCR1. Data are average of three independent experiments and error bars represent ± SD. Product profiles of BLC (open) and BLCR1 (filled symbols) are shown.

By adding 1 g/L of acetaldehyde to the medium, the effect of acetaldehyde on TMP and acetoin generation by BLC was explored. The TMP and acetoin production were also impacted by acetaldehyde supplementation of the BLC fermentation medium (Fig. 5). Relative to unsupplemented BLC, maximal TMP and acetoin yields were improved from 43.75 g/L to 47.26 g/L (an increase of 8.1% (w/w) and from 18.73 g/L to 20.13 g/L (an increase of 7.5% (w/w) respectively in BLCR1 (BLC with 1 g/L acetaldehyde). When the two recombinant strains were grown for 144 h and 168 h, these increases were observed (Fig. 5). As such, 1 g/L is also an optimal starting acetaldehyde concentration for achieving maximal TMP and acetoin by recomibniant BLC strain. However, there was almost no change in the yield of 2,3-BD.

## Discussion

**Figure 6:**
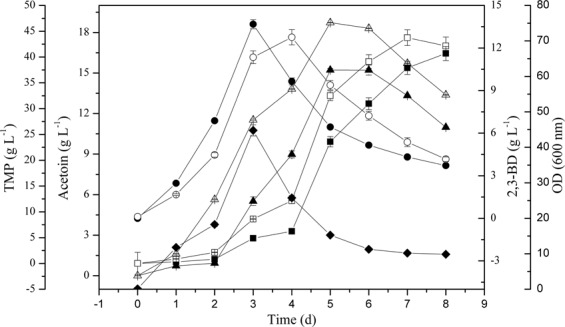
Characterization of the *aldC*-over expressed transformants. Data are average of three independent experiments and error bars represent ± SD. Effects of aldC-overexpression on acetoin, TMP, and 2,3-BD production during the stationary phase of cultivation with 200 ml LB in 500 ml flask shaken at 200 rpm and 37 °C for 8 day. Product profiles of BL1 (filled symbols) and BLC (open) are shown. Squares, triangles, and circles represent TMP, acetion, and 2,3-BD concentrations of the same strain, respectively.

When the recombinant strains were grown for 168 h and 120 h respectively, the yield of TMP and acetoin in recombinant BLC rose by 23.99% (w/w) and 28.98% (w/w) compared with *Bacillus lincheniformis* BL1. The yield of TMP and acetoin in recombinant BLS increased slightly at the same time periods (Fig. 3). Compared with *Bacillus lincheniformis* BLC, the yield of TMP and acetoin in *Bacillus lincheniformis* BLCS decreased slightly with 2,3-BD increased slightly (Fig. 3). This rise was primarily attributable to (i) a lack of de novo early stationary phase acetaldehyde production, and (ii) acetoin precursor accumulation during this same time period. When cultured for 72 h and 120 h, BL1 accumulated over 13.6 g/L of 2,3-BD and 15.2 g/L of acetoin, respectively. The aldC overexpressed mutant strain (BLC) accumulated less than 11.4 g/L 2,3-BD and 18.8 g/L acetoin when cultured for 72 h and 120 h, respectively, in media containing 0.96 g/L residual glucose. Although acetoin and 2,3-BD levels fell during the stationary phase (Fig. 6). A prior study has similarly found that there is a 2,3-BD degradation pathway in which acetoin functions as an intermediate^18^. In contrast, the production of 2,3-BD in recombinant BLS was more substantial than those of BL1 and BLC (Fig. 3).

After 120 h, acetoin levels in BL1 samples began to fall as a consequence of ongoing degradation (Fig. 6). Acetoin can serve as a carbon source or a TMP precursor for BL1^19^. The concentration of acetoin was increased in BLC; resulting in a higher TMP concentration. However, BL1 can metabolize acetoin more rapidly than can BLC^20^.

**Figure 7:**
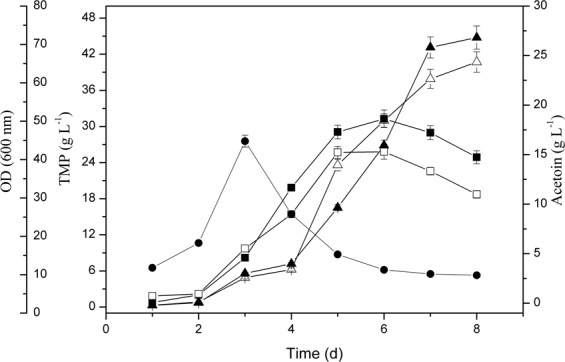
Effect of acetaldehyde addition on Acetoin production (■) and TMP production (▲) by *B. lincheniformis* BL1. Data are average of three independent experiments and error bars represent ± SD. Product profiles of BL1 (open) and BL1R1 (filled symbols) are shown.

Interestingly, with the acetaldehyde concentration in BL1 media rose from 0 g/L to 1 g/L, TMP and acetoin yields similarly rose from 37.89 g/L to 44.77 g/L and from 15.22 g/L to 17.94 g/L, respectively. Thus, acetaldehyde can facilitate TMP and acetoin production in a dose-dependent fashion. A 1 g/L acetaldehyde concentration was sufficient to achieve maximal TMP and acetoin yields. Nevertheless, the mechanism that the improvement of acetoin with acetaldehyde supplementation is poorly understood. We have hypothesized from the existing acetoin cleavage pathway that degradation of acetoin in microbial cells occurs on two levels. The first is the reversible transformation between acetoin and 2,3-BD. Secondly, acetoin can be used to produce acetyl-CoA and acetaldehyde under the action of the acetoin dehydrogenase complex (Ao DH ES), and acetaldehyde can then be converted into acetic acid or ethanol. Thus, the acetoin dehydrogenase system can catalyze the conversion of acetoin to acetaldehyde, and 2,3-BD dehydrogenase or acetoin reductase likely catalyzes the conversion between 2,3-butanediol (2,3-BD) and acetoin^21^. Therefore, in our study, the initial acetaldehyde may have a feedback inhibition effect on the catalytic conversion of acetoin into acetaldehyde, which raises the concentration of acetoin and 2,3-BD, and also enhances the ability to synthesize TMP (Fig. 4). Alternatively, the initial addition of acetaldehyde could have a feedback inhibition effect on the Ao DH ES, which affects the conversion of acetoin into acetaldehyde and results in the accumulation of acetoin production, enhancing the ability to synthesize TMP in turn. In addition, the initial acetaldehyde can enhance the metabolism of acetaldehyde to acetyl-CoA, which indirectly provides feedback inhibition of pyruvate to the acetyl-CoA metabolic branch, thereby enhancing the pyruvate to acetoin metabolic branch (Fig. 2). So, when the acetaldehyde was added into the medium, there was an accumulation of the precursor acetoin in BL1 or BLC and an increased yield of TMP (Figs. 5 and 7). Acetaldehyde is less favorable for cell growth (Fig. 4). Enzymatic activity is most robust when high levels are carbohydrates are available, and they fall once these carbon sources are exhausted^22^. Thus, the initial suppression of the acetoin dehydrogenase system by acetaldehyde can impede the synthesis of acetaldehyde when remaining glucose levels at the end of fermentation were minimal. There is further evidence suggesting that 2,3-BD can be utilized as a carbon source in order to produce acetoin in the context of low carbon availability^14^. However, cell growth will be adversely affected, and the product synthesed also be inhibited (Table 3) when the specific inhibitory concentration of acetaldehyde reached. The acetaldehyde inhibitory mechanisms is still unclear, suggesting that there may be certain differentially regulated enzymes that can be impacted by acetaldehyde and which are involved in metabolic or synthetic processes, or the transfer process could be induced by different enzymes. Further study of the inhibitory role of acetaldehyde is thus warranted.

In summary, we constructed a recombinant BLC strain for producing then high tetramethylpyrazine (TMP). Altering acetoin biosynthetic pathway carbon flux can effectively improve TMP yields. The overexpression of α-acetolactate decarboxylase (*aldC*) enhanced the strength of pathways responsible for competition and acetoin catabolism, which during the early stationary phase resulted in precursor acetoin accumulation and impaired 2,3-BD production. In flask fermentation, Compared with BL1 strain, the yield of TMP and acetoin in BLC rose by 23.99% and 28.98%, respectively. The addition of different concentrations of acetaldehyde enhanced TMP and acetoin production by BL1. Using acetaldehyde to supplement the substrate used for fermentation represents a novel means of readily enhancing TMP and acetoin production. We found that maximal TMP and acetoin yields rose by 13.83% (w/w) and 22.27 (w/w), respectively, when the acetaldehyde concentration were raised from 0 to 1 g/L. The underlying mechanisms should be further investigated.

### Ethics approval and consent to participate

Studies with human participants or animals performed by any of the authors were not contained in this manuscript.

## Data Availability

All relevant data analyzed or generated during this study were included in this published article.

## References

[1] Xiao ZJ, Xie NZ, Liu PH, Hua DL, Xu P (2006). Tetramethylpyrazine production from glucose by a newly isolated Bacillus mutant. Appl. Microbiol. Biotechnol..

[2] Muller R, Rappert S (2010). Pyrazines: occurrence, formation and biodegradation. Appl. Microbiol. Biotechnol..

[3] Fan W, Xu Y, Zhang Y (2007). Characterization of pyrazines in some chinese liquors and their approximate concentrations. J. Agr. Food. Chem..

[4] Ai-ping Z, Di-xun W, Feng W (1986). Effect of tetramethylpyrazine on acute and chronic hypoxic pulmonary hypertension of the rat. J. Tongji. Med. Univ..

[5] Lancker FV, Adams A, Kimpe ND (2010). Formation of pyrazines in maillard model systems of lysine-containing dipeptides. J. Agr. Food. Chem..

[6] Lancker FV, Adams A, Kimpe ND (2012). Impact of the N-terminal amino acid on the formation of pyrazines from peptides in maillard model systems. J. Agr. Food. Chem..

[7] Hao F, Wu Q, Xu Y (2013). Precursor supply strategy for tetramethylpyrazine production by *Bacillus Subtilis* on solid-state fermentation of wheat bran. Appl. Biochem. Biotechnol..

[8] Amrani-Hemaimi M, Cerny C, Fay LB (1995). Mechanisms of formation of alkylpyrazines in the maillard reaction. J. Agr. Food. Chem..

[9] Zhu BF, Xu Y, Fan WL (2010). High-yield fermentative preparation of tetramethylpyrazine by *Bacillus sp*. using an endogenous precursor approach. J. Ind. Microbiol. Biotechnol..

[10] Lancker FV, Adams A, Kimpe ND (2011). Chemical modifications of peptides and their impact on food properties. Chem. Rev..

[11] Kosuge T, Adachi T, Kamiya H (1962). Isolation of tetramethylpyrazine from culture of bacillus natto, and biosynthetic pathways of tetramethylpyrazine. Nature..

[12] Xu Y, Wu Q, Fan WL, Zhu BF (2011). The discovery and verification of the production pathway of Tetramethylpyrazine (TTMP) in chiese liquor. Liquor-Making. Sci. Technol..

[13] Wu JF, Yan X (2014). Formation mechanism of tetramethylpyrazine produced with *B. subtilis* S12 under the fermentation condition simulated bacterial qu preparation used for chinese liquor brewing. J. Food. Sci. Biotechnol..

[14] Zhang X (2011). Isolation and identification of an acetoin high production bacterium that can reverse transform 2,3-butanediol to acetoin at the decline phase of fermentation. World. J. Microb. Biotechnol..

[15] Sabik H, Fortin J, Martin N (2012). Identification of pyrazine derivatives in a typical maple syrup using headspace solid-phase microextraction with gas chromatography–mass spectrometry. Food. Chem..

[16] Gao S (2014). Characterization of acetoin production in a *budC* gene disrupted mutant of Serratia marcescens G12. J. Ind. Microbiol. Biotechnol..

[17] Shi L, Gao S, Yu Y, Yang H (2014). Microbial production of 2,3-butanediol by a newly-isolated strain of *Serratia marcescens*. Biotechnol. Lett..

[18] Thanh T (2010). Regulation of acetoin and 2,3-butanediol utilization in *Bacillus licheniformis*. Appl. Microbiol. Biotechnol..

[19] Huang M, Oppermann-Sanio FB, Steinbuchel A (1999). Biochemical and molecular characterization of the Bacillus subtilis acetoin catabolic pathway. J. Bacteriol..

[20] Silbersack J (2006). An acetoin-regulated expression system of *Bacillus subtilis*. Appl. Microbiol. Biotechnol..

[21] Juni E, Heym GA (1956). A cyclic pathway for the bacterial dissimilation of 2,3-butanediol, acetylmethyl carbinol and diacetyl II.: The synthesis of diacetylmethylcarbinol from diacetyl, a new diphosphothiamin catalyzed reaction. J. Bacteriol..

[22] Skory CD (2000). Isolation and expression of lactate dehydrogenase genes from Rhizopus oryzae. Appl. Environ. Microb..

